# Interindividual variability in platelet reactivity among individuals with or without antiplatelet therapy: results from a large tertiary care hospital

**DOI:** 10.1007/s11239-024-03022-w

**Published:** 2024-09-06

**Authors:** Mattia Galli, Sergio Terracina, Eleonora Schiera, Massimo Mancone, Luigi Frati, Dominick J. Angiolillo, Fabio M. Pulcinelli

**Affiliations:** 1 Maria Cecilia Hospital, GVM Care & Research, Cotignola, Italy; 2 Department of Experimental Medicine, Sapienza University of Rome, Viale Regina Elena 324, Rome, 00161 Italy; 3 Department of Clinical Internal, Anesthesiological and Cardiovascular Sciences, Sapienza University of Rome, Rome, Italy; 4 IRCCS Neuromed, Pozzilli, IS Italy; 5 Division of Cardiology, University of Florida College of Medicine, Jacksonville, FL USA

**Keywords:** Platelet aggregation, Aspirin, Clopidogrel, Antiplatelet therapy

## Abstract

Antiplatelet therapy is crucial for reducing thrombotic events in patients with atherosclerotic disease, but the response vary widely among individuals. The identification of patients at high (HPR), optimal (OPR) or low platelet reactivity (LPR) is dependent on high interlaboratory variability. We report results of a large dataset of patients to assess the gold standard light transmission aggregometry (LTA). A total of 11,913 patients who sequentially underwent LTA assessment using several stimuli (ADP-2µM, collagen-2 µg/ml, arachidonic acid 0.5 mM, epinephrine 10µM) with a standardized methodology between 2004 and 2022 were screened. After application of inclusion-exclusion criteria, 5,901 patients were included and divided into five groups: healthy-volunteers (HV; *N* = 534); controls (CTR; *N* = 1073); aspirin-treated patients (ASA; 75–150 mg/die; *N* = 3280); clopidogrel-treated patients (CLOP; 75 mg/die; *N* = 495) and patients treated with dual antiplatelet therapy, ASA plus CLOP (DAPT; *N* = 519). The mean PA% in response to ADP 2 μm was 72.4 ± 33.3 in the CTR population, 40.6 ± 29.9 in the ASA group, 25.1 ± 35.1 in the CLOP group and 10.2 ± 18.5 in the DAPT group. The mean PA% in response to collagen 2 ug/ml was 90.7 *±* 10.5 in the CTR population, 40.8 ± 26.3 in the ASA group, 79.4 ± 21.8 in the CLOP group and 17.9 ± 19.9 in the DAPT group. The percentage of patients at OPR following ADP stimuli was 66%, 25%, and 26%, in the ASA, CLOP, and DAPT group, respectively. The percentage of patients at OPR following collagen stimuli was 56%, 22%, and 41%, in the ASA, CLOP, and DAPT group, respectively. LTA was significantly increased in response to ADP (72.4 ± 33.3vs62.7 ± 37.1; *p* < 0.001) and AA (90.7 ± 15.6vs87.6 ± 20.5; *p* < 0.001) in CTR compared to HV. Our findings support the concept that a significant proportion of individuals present a hyper- or hypo-reactive platelet phenotype potentially affecting the safety and efficacy of antiplatelet therapy. The variability in response to antiplatelet therapy was particularly evident in patients undergoing single as opposed to dual antiplatelet therapy regimens. These data support ongoing strategies of guided selection of antiplatelet therapy in patients with cardiovascular disease.

**Graphical Abstract:**

**Figure:**
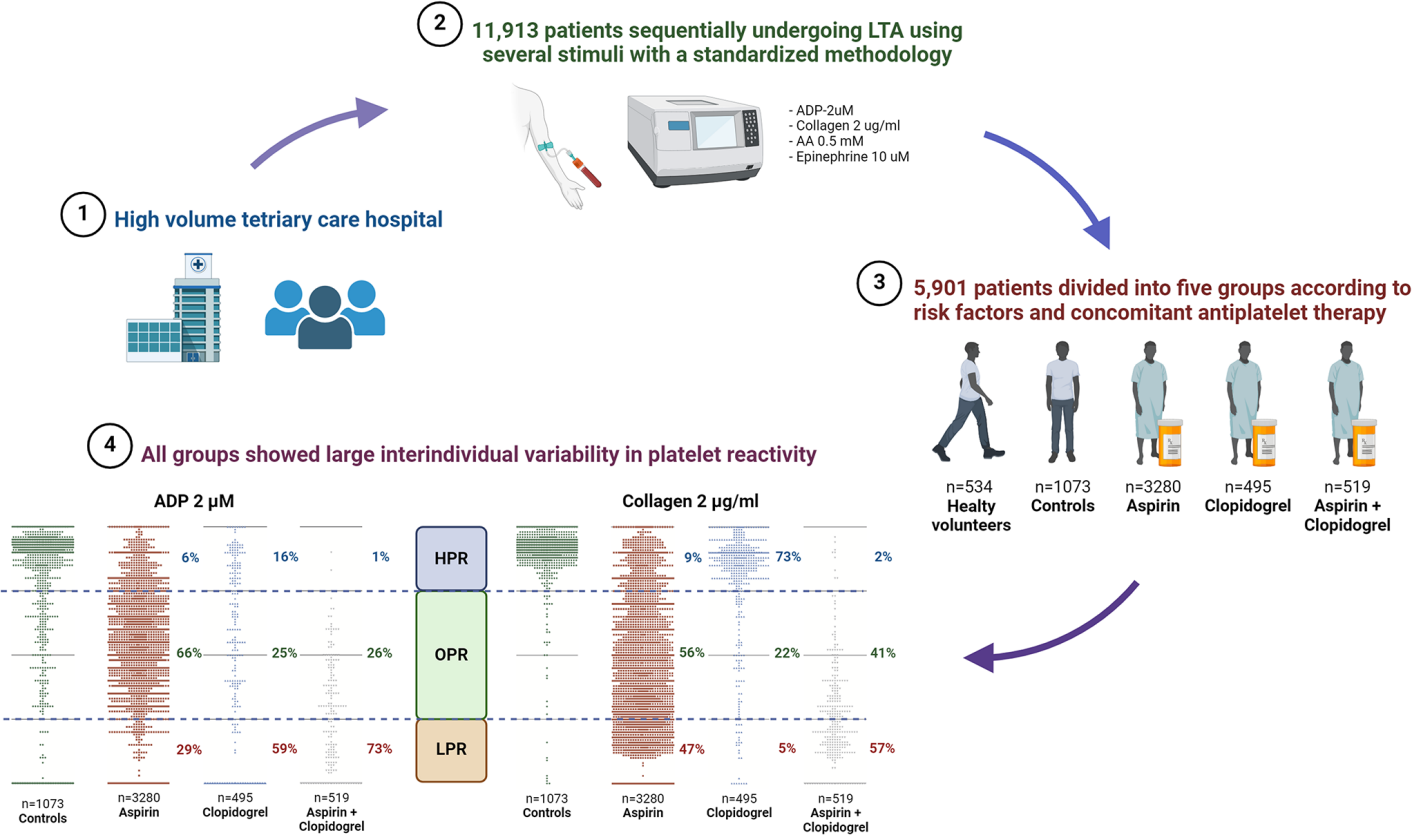
Inter-individual variability in platelet reactivity among controls and patients treated with antiplatelet therapy. A total of 5901 patients were selected from 11,913 subjects who underwent LTA with standardized methodology in our high-volume tertiary care hospital. The selected patients were divided into 5 groups based on risk factors and antiplatelet treatment. We present the scatter dot plots of patients belonging to the CTR, ASA, CLOP, and DAPT populations tested with ADP 2µM (left) and collagen 2 µg/ml (right). OPR, LPR and HPR platelet reactivity were defined using PA %: patients with PA between 25% and 75% were considered OPR, those with PA% ≤ 24% were considered LPR and those with PA ≥ 76% were considered HPR. Less than 2/3 of treated patients were at OPR. Controls (patients with at least one cardiovascular risk factor) also showed a large variability in platelet reactivity. CTR, control group; ASA, aspirin group; CLOP, clopidogrel group; DAPT, double antiplatelet therapy group; OPR, optimal platelet reactivity; HPR, high platelet reactivity; LPR, low platelet reactivity; PA, platelet aggregation

**Supplementary Information:**

The online version contains supplementary material available at 10.1007/s11239-024-03022-w.

## Highlights

Guided selection of antiplatelet therapy reduced ischemic events without any trade-off in bleeding, but, the optimal assessment of the “therapeutic response” to antiplatelet therapy is unclear.

A total of 11,913 patients, allocated in several groups (healthy volunteer, control, aspirin, clopidogrel and double antiplatelet therapy), platelet aggregation with a standardized methodology was studied.

A wide inter-individual variability in platelet function was found in patients of all groups studied.

Individuals with cardiovascular risk factors showed increased platelet aggregation compared with individuals without any risk factor.

Platelet aggregation may be a useful tool to study platelet reactivity in a strategy of guided escalation or de-escalation of antiplatelet therapy to optimize clinical outcomes.

**Supplementary Information:**

The online version contains supplementary material available at 10.1007/s11239-024-03022-w.

## Introduction

Antiplatelet therapy is crucial for reducing thrombotic events in patients with atherosclerotic disease [1]. In particular, dual antiplatelet treatment (DAPT) with aspirin and a P2Y_12_ inhibitor (clopidogrel, prasugrel or ticagrelor) is the standard of care in high-risk patients such as those with acute coronary syndromes (ACS) or undergoing percutaneous coronary interventions (PCI) [1]. Nevertheless, the response to antiplatelet agents widely changes among individuals, and the reduction of thrombotic events may come at the expenses of increased bleeding [2, 3]. Pharmacodynamic (PD) investigations have shown that the degree of platelet inhibitory effects exerted by antiplatelet agents correlates with adverse outcomes [4, 5]. In particular, patients with high (HPR) and low platelet reactivity (LPR) while treated with antiplatelet therapy are at increased risk of thrombotic and bleeding events, respectively [5, 6]. These observations have prompted investigations assessing the impact of platelet function testing (PFT) as a strategy to guide the selection of antiplatelet drug regimens to optimize safety and efficacy outcomes [7].

A recent meta-analysis carried out on a sample of over 60,000 patients showed that a guided selection of antiplatelet therapy, is associated with improved composite and individual efficacy outcomes with a favorable safety profile driven by a reduction in bleeding, compared with a standard selection [8]. Moreover, the use of a guided selection of antiplatelet therapy has shown to improve outcomes regardless of the clinical setting and the antiplatelet strategy used as comparator arm. Specifically, a guided selection of antiplatelet therapy reduced ischemic events without any trade-off in bleeding (i.e., escalation strategy) when compared to a standard DAPT with clopidogrel in chronic coronary syndromes and reduced bleeding without any trade-off in ischemic events (i.e. de-escalation strategy) compared to DAPT with prasugrel or ticagrelor in ACS [5, 9]. However, a key challenge to the implementation of PFT to guide the selection of antiplatelet therapy is that platelet aggregation (PA) is highly dependent on pre-analytical and analytical variables leading to high interlaboratory variability. We here report the results of a large dataset of patients on different antiplatelet treatment regimens from a high-volume single center laboratory using a standardized methodology to assess PA and identify patients with HPR, LPR, or optimal platelet reactivity (OPR).

## Materials and methods

This is a retrospective analysis conducted on 11,913 subjects who sequentially underwent PFT at Policlinico Umberto I University Hospital, UP Advanced Diagnosis Platelet Disorders (Rome, Italy) between January 1, 2004 and December 31, 2022. The population was divided into five groups: (1) healthy volunteers (HV), consisting in subjects with no cardiovascular risk factors, without any chronic diseases and not taking any therapy for two or more weeks before blood testing; (2) controls (CTR), with at least one cardiovascular risk factor (hypertension, diabetes, hypercholesterolemia, previous thrombotic event) but not treated with antiplatelet therapy; (3) patients treated with low-dose aspirin (ASA; 75–150 mg/die); (4) patients treated with clopidogrel (CLOP; 75 mg/die); and (5) patients treated with dual antiplatelet therapy (DAPT) consisting of ASA plus CLOP.

Patients treated with antiplatelet therapy required to on treatment for at least one month before PA assessment. Compliance to antiplatelet therapy was evaluated by face-to-face interview carried out by healthcare staff. Among CTR patients and those on antiplatelet therapy, subjects were excluded if any of the following were present: comorbidities associated with abnormal platelet function, non-compliance, concomitant use of drugs interfering with platelet function, doses of aspirin and clopidogrel different from those required to be included in the study. Detailed inclusion and exclusion criteria are reported in the Supplementary appendix.

### Blood sampling and laboratory assessments

The assessment of PA may be affected by multiple variables resulting in data variability, we used a standardized methodology that is reported in detail in the Supplementary appendix. In brief, light transmission aggregometry (LTA) following Scientific and Standardization Committee/International Society on Thrombosis and Haemostasis (SSC/ISTH) subcommittee on Platelet Physiology recommendations was used, paying particular attention to the pre-analytical and sample preparation variables [10].

Pre-clinical variables are reported in the supplement and briefly included: blood samples drawn into plastic tube (Greiner Bio-one, North America, Inc.) of 129 mM sodium citrate and the analysis completed within a maximum of 4 h after blood sampling. Sample preparation variables included: (1) Platelet-rich plasma (PRP) prepared by centrifuging blood samples at 200 g for 15 min at RT without using a brake; (2) Platelet-poor plasma (PPP) prepared by centrifuging whole blood at ambient temperature at 2000 g for 10 min; (3) PRP stays at room temperature at least 15 min before testing.

### Platelet agonists

In order to provide a comprehensive evaluation of PA, we used the following platelet agonists: adenosine-diphosphate (ADP), collagen, epinephrine, and arachidonic acid (AA). Specifically:

- ADP (Helena Biosciences Europe, Gateshead, United Kingdom): we analyzed ADP at the concentration of 2 µM.
- Collagen (Mascia Brunelli, Milano Italy): we analyzed collagen at the concentration of 2 µg/ml.
- Epinephrine (Helena Biosciences Europe, Gateshead, United Kingdom): at concentrations of 10 µM.
- AA (Helena Biosciences Europe, Gateshead, United Kingdom): AA was analyzed at concentrations of 0.5 mM in HV, CTR and CLOP patients and with a concentration of 0.75 mM in ASA and DAPT patients.

PA was monitored for 5 min after adding an agonist in HELENA Apact 4 Apparatus (Helena Biosciences Europe, Gateshead, United Kingdom) The results were reported as percentage of platelet aggregation (PA%) obtained at 4 min.

### Study endpoints

The analysis consisted in three different parts. First, we compared PA of patients on ASA, CLOP or DAPT with PA from the CTR group. Indeed, the CTR group was identified as the most reliable for a comparison with patients treated with single antiplatelet drug or DAPT, minimizing the differences in baseline characteristics potentially affecting PA (i.e., co-treatment or comorbidities). Second, we compared PA assessed by mean ± Standard Deviation (SD) in the HV versus CTR group. Third, we aimed at identifying the proportion of patients among different groups with OPR, LPR and HPR using PA %. Specifically, patients with PA% between 25 and 75 were considered OPR, while those with PA% < 25% were considered LPR and those with PA > 75% were considered HPR. The choice to use PA% to classify patients in OPR, LPR and HPR was reported in the supplement [11].

### Statistical analysis

Continuous variables are presented as mean ± SD of the PA%. Categorical variables of patients are expressed as frequencies and percentages. Comparisons between categorical variables was performed using 2 tailed Fisher’s exact test or the Pearson’s chi square test. Continuous variables were presented as mean ± SD. Student t test and Mann–Whitney U test were used to compare continuous variables according to normal distribution tested by the Kolmogorov–Smirnov test. Data were also reported as median and 25°(Q1) and 75° (Q3) percentiles (IQR) values of the PA% at 4 min and presented using tables and scatter dot plots. Differences were calculated using Wilcoxon’s test for unpaired data. A *P*-value < 0.05 was considered statistically significant. All analyses were performed using GraphPad Prism 9, version 9.1.0.

## Results

**Fig. 1:**
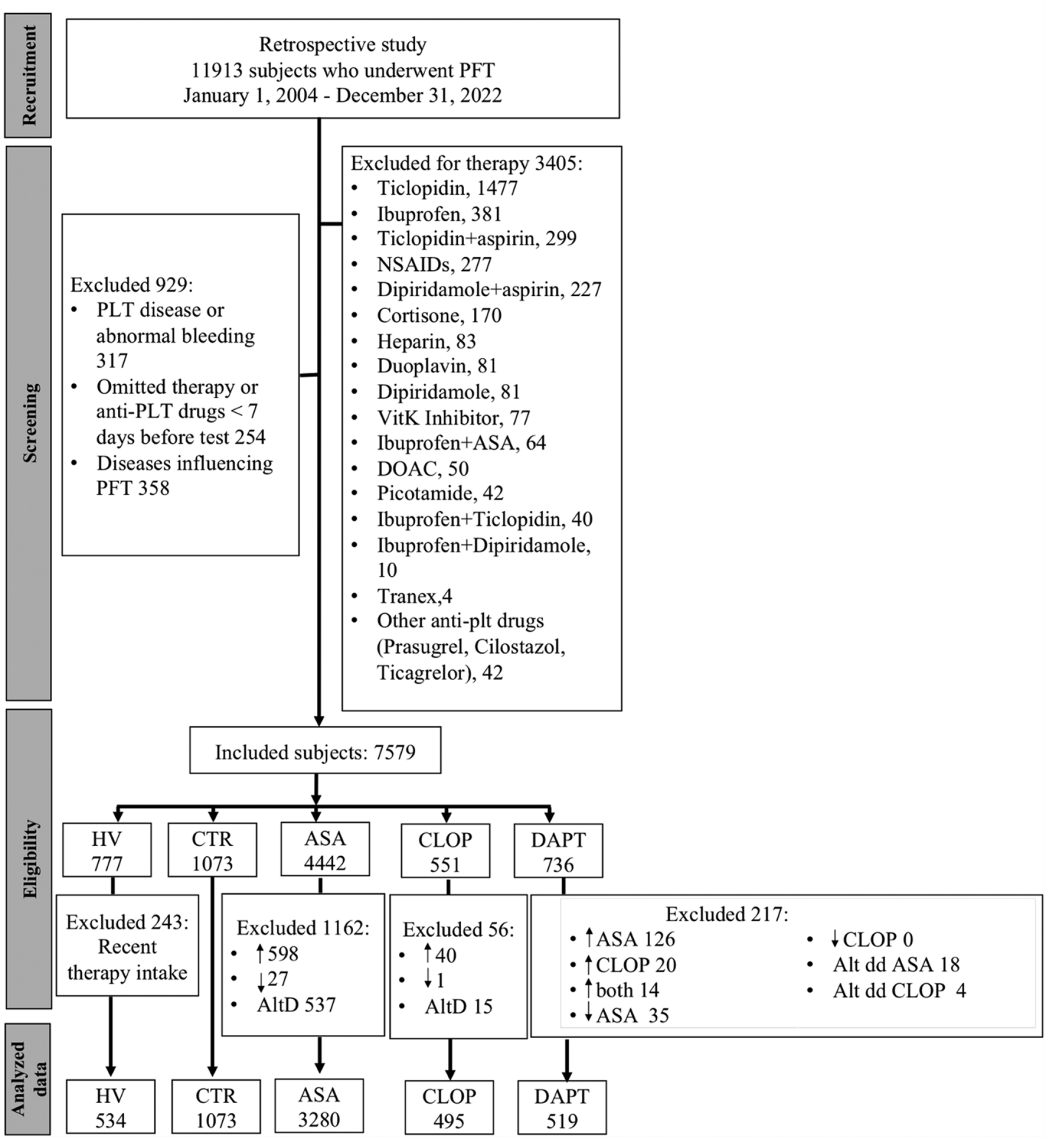
Flow diagram. A total of 7,579 subjects were included from the original group of 11,913. The remaining patients were allocated into 5 different groups: (1) HV group (*n* = 777), 243 of which were excluded because taking therapies affecting platelet function; (2) CTR group (*n* = 1073); (3) ASA group (*n* = 4442), 1162 of which were excluded because taking higher (↑, > 150 mg) or lower than recommended aspirin dosage (↓, < 75 mg), or taking it at alternate days (AltD); (4) CLOP group (*n* = 551), 56 of which were excluded because taking higher (↑, > 75 mg) or lower than recommended clopidogrel dosage (↓, < 75 mg), or taking it at alternate days (AltD); (5) DAPT group (*n* = 736), 217 of which were excluded for the same reasons as above. Alternate days therapy, AltD; Aspirin, ASA; clopidogrel, CLOP; Control, CTR; Dual antiplatelet therapy, DAPT; direct oral anticoagulants, DOAC; healthy volunteers, HV; non-steroidal anti-inflammatory drugs, NSAIDs; vitamine K, VitK; platelet function testing, PFT; platelet, PLT, ↑ higher dosage, ↓ lower dosage

A total of 5901 patients met study entry criteria: (1) HV (*n* = 534); (2) CTR (*n* = 1073); (3) ASA patients (*n* = 3280); (4) CLOP patients (*n* = 495); (5) DAPT patients (*n* = 519). A flow diagram of patient selection is illustrated in Fig. 1. Baseline characteristics and concomitant medications of patients in each group are reported in Table 1. There were no differences in baseline characteristics between the CTR and treatment groups in terms of risk factors and therapies used, with the sole exception of history of coronary artery disease or myocardial infarction and of previous stroke/transient ischemic attack that was more frequent in the DAPT and CLOP group, respectively. Conversely, the HV population comprises subjects without risk factors, no other diseases and who didn’t receive any medication in the previous 15 days before blood sampling. The major difference between HV and CTR population was the lower mean age (43±21 and 64±13, respectively).

**Table 1:** Demographic and clinical characteristic of the study population of healthy volunteers (HV), aspirin and clopidogrel free control population (CTR), patients on aspirin therapy (ASA), patients on clopidogrel therapy (CLOP), patients on aspirin + clopidogrel therapy (DAPT). Angiotensin-converting enzyme, ACE; angiotensin receptor blockers, ARBs; coronary artery disease, CAD; myocardial infarction, MI; peripheral artery disease, PAD; standard deviation, SD; transient ischemic attack, TIA

Patient characteristics	Patients groups				
	HV (*N* = 534)	CTR (*N* = 1073)	ASA (*N* = 3280)	CLOP (*N* = 495)	DAPT (*N* = 519)
Age (mean ± SD, y)	43 ± 21	64 ± 13	68 ± 12	72 ± 9	66 ± 13
Males, n (%) Females, n (%)	176 (33) 358 (67)	343 (32) 730 (68)	1254 (38) 2026 (62)	185 (37) 310 (63)	345(66) 174 (34)
**Clinical conditions and risk factors (RF)**					
Hypertension, n (%)	0 (0)	662 (62)	1939 (59)	329 (67)	283 (55)
Hypercholesterolaemia, n (%)	0 (0)	394 (37)	1252 (38)	230 (47)	240 (46)
Smoking, n (%)	0 (0)	229 (21)	444 (14)	99 (20)	30 (6)
Diabetes, n (%)	0 (0)	110 (10)	309 (9)	64 (13)	39 (7)
CAD/MI, n (%)	0 (0)	36 (3)	262 (8)	57 (12)	327 (63)
PAD, n (%)	0 (0)	11 (1)	31 (1)	2 (0)	1 (0)
Previous stroke/TIA, n (%)	0 (0)	297 (28)	856 (26)	254 (51)	50 (10)
Retinal thrombosis, n (%)	0 (0)	14 (1)	60 (2)	9 (2)	1 (0)
Carotid obstruction, n (%)	0 (0)	64 (6)	234 (7)	40 (8)	19 (4)
**Medications**					
Beta-blocking agents, n (%)	0 (0)	174 (16)	559 (17)	102 (21)	144 (28)
Calcium channel blocker, n (%)	0 (0)	179 (17)	580 (18)	100 (20)	55 (11)
ACE-I, n (%)	0 (0)	197 (18)	742 (23)	83 (17)	122 (24)
ARBs/angiotensin 2, n (%)	0 (0)	225 (21)	703 (21)	142 (29)	68 (13)
Nitrates, n (%)	0 (0)	13 (1)	127 (4)	20 (4)	46 (9)
Statin, n (%)	0 (0)	217 (20)	966 (29)	187 (38)	205 (39)
Antidiabetic drugs, n (%)	0 (0)	101 (9)	256 (8)	61 (12)	26 (5)
Diuretic, n (%)	0 (0)	60 (6)	255 (8)	36 (7)	43 (8)
Omega-3, n (%)	0 (0)	37 (3)	145 (4)	43 (9)	75 (14)

### Platelet aggregation in patients with single or dual antiplatelet therapy compared to controls

#### ADP

The mean±SD of PA% in response to ADP 2 µM was 72.4±33.3 in the CTR population, 40.6±29.9 in the ASA group, 25.1±35.1 in the CLOP group and 10.2±18.5 in the DAPT group.

Table S1 reports mean±SD and median (IQR) of each group. CLOP, and particularly DAPT, were the two treatments associated with the greatest reduction of platelet function induced by ADP.

**Fig. 2:**
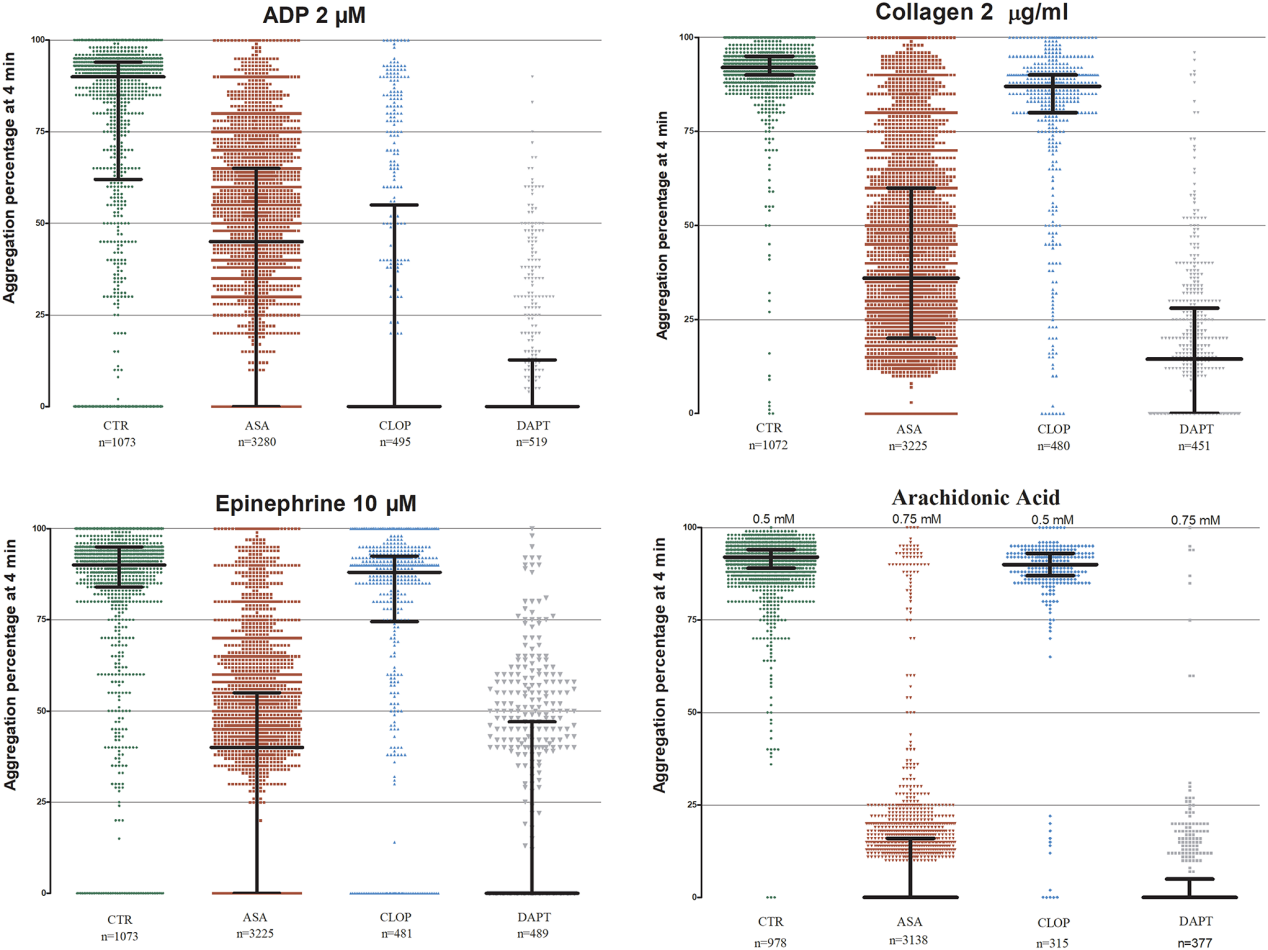
Scatter dot plot of each patient belonging CTR, ASA, CLOP, DAPT populations tested with ADP 2µM. (**A**); Collagen 2 µg/ml (**B**); Epinephrine 10µM (**C**); AA 0.5 mM for CTR and CLOP populations; ASA and DAPT populations tested with AA 0.75 mM (**D**). Lines represent median with IQR range. N, number of patients analyzed. CTR, control group; ASA, aspirin group; CLOP, clopidogrel group; DAPT, double antiplatelet therapy group; IQR, interquartile range

In fact, mean PA% in response to ADP 2µM was significantly reduced in the CLOP group compared with the CTR population (25.1 ± 35.1 vs. 72.4 ± 33.3; *p* < 0.0001) and with DAPT compared with the CTR population (10.2±18.5 vs. 72.4 ± 33.3; *p* < 0.0001). Moreover, the median PA% in CTR patients was 90%, whereas within the CLOP population 61% of them showed absence of response (Fig. 2). Although with a more modest effect compared with CLOP-based treatments, ASA significantly reduced mean PA% compared with the CTR population (40.6±29.9 vs. 72.4 ± 33.3; *p* < 0.0001). Moreover, the median PA% was 45% with ASA compared with 90% of the CTR population (*p* < 0.0001).

#### Collagen

The mean±SD of PA% in response to collagen 2 µg/ml was 90.7±10.5 in the CTR population, 40.8±26.3 in the ASA group, 79.4±21.8 in the CLOP group and 17.9±19.9 in the DAPT group (Fig. 2). Table S1 reports mean±SD and median (IQR) of each group. ASA and DAPT were the two treatments associated with the greatest reduction of platelet function induced by collagen. In fact, mean PA% in response to collagen at concentration 2 µg/ml was significantly reduced in the ASA group compared with the CTR population (40.8±26.3 vs. 90.7±10.5; *p* < 0.0001) and with DAPT compared with the CTR population (17.9±19.9 vs. 90.7±10.5; *p* < 0.0001). Moreover, the median PA% was 92% in CTR patients whereas was 36% and 15% in the ASA and DAPT group, respectively. Although with a more modest effect compared with ASA-based treatments, CLOP significantly reduced mean PA% compared with the CTR population (79.4±21.8 vs. 90.7±10.5; *p* < 0.0001). Moreover, the median PA% was 87% with CLOP compared with 92% of the CTR population.

#### Epinephrine

The mean±SD of PA% in response to epinephrine 10 µM was 82.5±23.4 in the CTR population, 32.1±30.8 in the ASA group, 74.8±31.1 in CLOP group, and 21.3±27.9 in the DAPT group (Fig. 2). Table S1 reports mean±SD and median (IQR) of each group. Similarly with collagen, the mean PA% in response to epinephrine 10 µM was significantly reduced in ASA group compared with CTR population (32.1±30.8 vs. 82.5±23.4; *p* < 0.0001) and with DAPT compared with CTR population (21.3±27.9 vs. 82.5±23.4; *p* < 0.0001). Moreover, the median PA% was 90% in CTR patients whereas was 40% and 0% in the ASA and DAPT group, respectively. Although with a more modest effect compared with ASA-based treatments, CLOP significantly reduced PA% compared with the CTR population (74.8±31.1vs 82.5±23.4; *p* < 0.0001). Moreover, the median PA% was 88% with CLOP compared with 90% of the CTR population.

#### Arachidonic acid

The mean±SD of PA% in response to AA 0.5 mM was 90.7±15.6 in the CTR population, and 86.5±16.7 in the CLOP group. Using higher concentration of AA (0.75 mM) in the ASA and DAPT groups, 78% and 76% of patients showed absence of PA, respectively (Fig. 2). Table S1 reports mean±SD and median (IQR) of each group. Notably, we found that in the ASA and DAPT groups, those with a PA > 20%, identified by other authors as resistant to the treatment (10), represent the 8% and 7% of entire population, respectively. CTR and CLOP patients showed similar mean±SD of PA% (90.7±15.6 and 86.5±16.7 respectively) and median PA% (92 and 90 respectively). Among ASA patients with AA-induced PA < 20%, 14% exhibited PA induced by collagen 2 µg/ml < 60% (Q3 of the ASA group). In the DAPT group, the corresponding values were 2% for collagen.

### Healthy volunteers versus controls

**Fig. 3:**
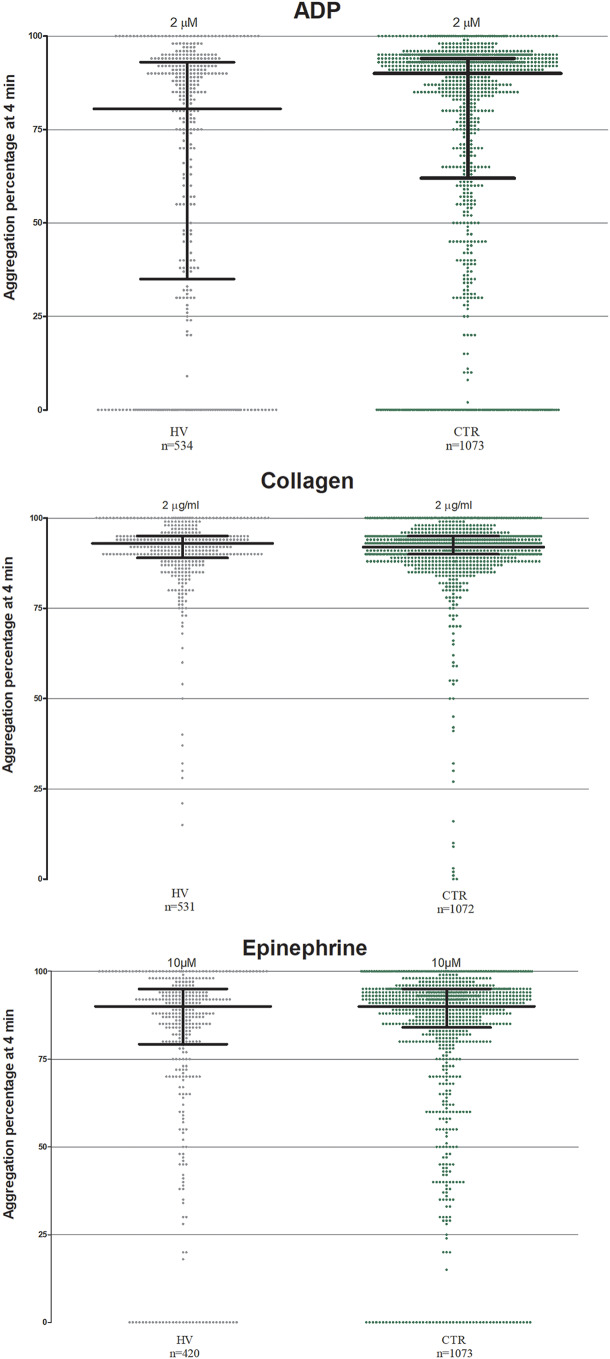
Scatter dot plot of each patients belonging HV and CTR populations tested with ADP 2µM (**A**); Collagen 2 µg/ml (**B**); Epinephrine 10µM (**C**). Lines represent median with IQR range. N = referred to number of patients analyzed. HV, healthy volunteers; CTR, control group; IQR, interquartile range

The mean±SD of PA% in CTR compared to HV individuals was significantly increased in response to ADP 2µM (72.4±33.3 and 62.7±37.1; *p* < 0.0001) and AA 0.5 mM (90.7±15.6 vs. 87.6±20.5; *p* < 0.0001) but not in response to collagen 2 µg/ml (90.7±11.6 vs. 90.7±10.5%; *p* = 0.47) or epinephrine 10µM (78.6±28.1 vs. 82.5±23.4; *p* = 0.58) (Fig. 3). Median PA% among CTR compared to HV individuals was significantly increased in response to ADP 2µM (90% vs. 80%; *p* < 0.0001) but not with AA 0.5 mM (92% vs. 93%), collagen 2 µg/ml (92% vs. 93%) or epinephrine 10µM (90% vs. 90%). Table S1 reports mean±SD and median (IQR) of each group.

### HPR, LPR and OPR among different groups

#### ADP

The percentage of patients with HPR (PA > 75%) was 6%, 16% and 1% in the ASA, CLOP, and DAPT group, respectively. The percentage of patients with LPR (PA < 25%) was 29%, 59% and 73%, in the ASA, CLOP and DAPT group, respectively. The percentage of patients with OPR (PA 25–75%) was 65%, 25% and 26%, in the ASA, CLOP, and DAPT group, respectively (Graphical Abstract).

#### Collagen

The percentage of patients with HPR (PA > 75%) was 9%, 73% and 2% in the ASA, CLOP, and DAPT group, respectively. The percentage of patients with LPR (PA < 25%) was respectively 47%, 5% and 57% in the ASA, CLOP and DAPT group. The percentage of patients with OPR (PA 25–75%) was 56%, 22% and 41% in the ASA, CLOP, and DAPT group, respectively (Graphical Abstract).

#### Epinephrine

The percentage of patients with HPR (PA > 75%) was 11%, 63% and 4% in the ASA, CLOP, and DAPT group, respectively. The percentage of patients with LPR (PA < 25%) was 30%, 10% and 56% in the ASA, CLOP and DAPT group, respectively. The percentage of patients with OPR (PA 25–75%) was 59%, 27% and 40% in the ASA, CLOP, and DAPT group, respectively.

#### Arachidonic acid

The percentage of patients with HPR (PA > 75%) was 2%, 54% and 1% in the ASA, CLOP, and DAPT group, respectively. The percentage of patients with LPR (PA < 25%) was 86%, 2% and 85%, in the ASA, CLOP and DAPT group, respectively. The percentage of patients with OPR (PA 25–75%) was 12%, 44% and 14% in the ASA, CLOP, and DAPT group, respectively. As reported above the AA concentration is different between the CLOP (0.5 mM) and ASA and DAPT populations (0.75 mM).

## Discussion

The main results of this large single-center study including patients undergoing PA assessment using a standardized methodology may be summarized as follows (**Graphical Abstract**): (1) the response to antiplatelet treatment is subject to wide inter-individual variability; (2) a substantial inter-individual variability in PA is also evident in HV and CTR; (3) aspirin-treated patients showed reduced PA in response to all stimuli, that was more pronounced in response to collagen, AA and epinephrine; (4) clopidogrel-treated patients showed reduced PA in response to all stimuli, that was more pronounced in response to ADP; (5) DAPT showed synergic effects in reducing PA compared to aspirin or clopidogrel alone in response to all the stimuli; (6) CTR showed increased PA in response to ADP compared with HV.

PA may be assessed by laboratory-based methods or point-of-care or near-patient-based [12]. Although point-of-care tests may represent a more practical option for patients with cardiovascular disease given the fact that they provide results in a more timely fashion and do not need expert personnel to be performed, laboratory-based methods remain the gold standard [5, 7, 12]. In particular, LTA represents the gold standard for the assessment of PA, but no single stimuli is reflective of global PA [13–15]. Indeed, thrombus formation is the result of the activation of several pathways that can be tested with LTA through the use of several agonists [13–15]. Unfortunately, the poor homogenization of results across laboratories represents an important limitation hindering the comparison of results across different [5]. A recent multicenter study, in which participated several laboratories, has highlighted that the adoption of some simple principles of standardization is effective in reducing the test variability between laboratories [12].

In our study we report the results of a large cohort of patients enrolled over the course of almost two decades, undergoing standardized laboratory assessments with the gold standard LTA and using a very pragmatic approach for defining on-treatment HPR, LPR or OPR based on PA% obtained. Indeed, the optimal cut-offs to be used to define OPR, LPR or HPR with LTA following different stimuli among patients undergoing antiplatelet therapy remains a topic of debate [11]. We defined patients with on-treatment OPR as those with PA% >25% and < 75%, while those with PA ≤ 24% were considered on LPR and those with PA ≥ 76% were considered HPR. Although this classification has been found to predict clinical outcomes in response to ADP in patients undergoing PCI treated with DAPT, its clinical impact with different stimuli among patients undergoing single antiplatelet therapy requires further investigations. However, this approach has the fundamental advantage of reducing inter-laboratory variability, providing important insights on the inter-individual variability in platelet reactivity among individuals.

Consistently with previous findings, we found that aspirin-based antiplatelet regimens primary affected LTA following stimuli with collagen, AA and epinephrine, while clopidogrel-based regimens primary affected LTA following ADP [14–17]. Moreover, DAPT was associated with synergistic effects on platelet reactivity reduction in response to all the stimuli, compared with ASA or CLOP alone [18]. Interestingly, we found that a substantial inter-individual variability in response to antiplatelet agents was present regardless the treatment regimen used, with only 12–66%, 22–44% and 14–41% of patients being classified as OPR in the ASA, CLOPI and DAPT groups, respectively, depending on the stimulus used for LTA. Of note, patients treated with DAPT were more often at LPR than those treated with ASA or CLOP alone, underlying the importance of the synergistic role of DAPT in reducing the rate of HPR, providing effective and predictable antiplatelet effects, but at the cost of increased risk of bleeding.

The large inter-individual variability in response to antiplatelet therapy has been particularly studied among patients undergoing DAPT with clopidogrel [6]. Indeed, it has been well established that 20–60% of clopidogrel treated patients is associated with reduced production of clopidogrel’s active metabolite leading to HPR and increased thrombotic events [5, 19, 20].

Although multiple mechanisms have shown to contribute to on-clopidogrel HPR, the hepatic cytochrome P450 (CYP) 2C19 enzyme plays a key role [3, 5, 19, 21, 22]. Indeed, CYP2C19 is crucial in the transformation of clopidogrel into its active metabolite, but carriers of loss-of function alleles for *CYP2C19* are relatively common and are characterized by reduced generation of clopidogrel’s active metabolite, increased rates of HPR and enhanced risk of thrombotic complications [5, 19, 21, 22]. Moreover, a number of studies have investigated the clinical impact of using alternative antiplatelet therapies among patients non-responding to clopidogrel [23, 24]. Finally, a guided selection of antiplatelet therapy, consisting in the implementation of PFT or genetic testing to selectively use alternative antiplatelet therapy among clopidogrel non-responders versus a standard antiplatelet therapy has been tested [25–28]. In particular, recent meta-analyses showed that a guided selection of antiplatelet therapy is associated with improved outcomes compared with a standard antiplatelet therapy when resulting both to a guide escalation or de-escalation of therapy [8, 9].

Our results confirm the large inter-individual variability in clopidogrel response using a standardized methodology based on LTA. The association between LPR and HPR with bleeding and ischemic events, respectively, supports the use of a guided selection of P2Y_12_ inhibiting therapy. Our results underline the need for guided therapy particularly in patients treated with clopidogrel monotherapy, a strategy that is increasingly used both in lieu of DAPT or in lieu of ASA for secondary prevention [29, 30].

Notably, the introduction of a guided P2Y_12_ inhibiting therapy potentially yield significant economic benefits. This impact is not solely due to the reduction in expenses linked to the utilization of more cost-effective and widely accessible medications like clopidogrel, but also stems from the improved outcomes and associated reduced need for hospitalization (15). These considerations are particularly important in those settings, such as developing countries, in which clopidogrel may be frequently used as a standard therapy also among ACS patients for economic reasons as well as reduced availability [23]. Furthermore, the cost of assessing PA using LTA is lower compared to other PFTs currently available.

With regards to patients treated with ASA, the reported prevalence of “aspirin resistance” vary widely between studies, ranging from 0 to 60% and being largely influenced by the aspirin dosage and the method used to define it [31–34]. In our database, there was no difference in platelet aggregation between 75 and 150 mg/die dosages (data not shown). Moreover, a study suggested that long-term treatment with aspirin may be associated with a progressive reduction in platelet sensitivity to this drug [35]. However, differently from patients non-responders to clopidogrel, the clinical impact of laboratory aspirin resistance and the advantage of using alternative antiplatelet therapies in these patients have not been univocally showed in clinical studies [36]. Our study confirms the inter-individual variability in aspirin response among individuals using a standardized methodology based on LTA, but the clinical implications of this laboratory variability remain to be determined.

Several studies have demonstrated antiplatelet treatment ability to reduce platelet reactivity, but all of them were conducted by evaluating differences in individual patients before and during treatment, without differentiating between categories of antiplatelet therapy [16, 37]. All these studies used a very small number of patients. Only one of our previous studies had performed aggregation in 176 aspirin-treated patients [35]. To the best of our knowledge, our study is the first one that, using a large number of patients, correlates platelet functionality using PA test between the populations undergoing antiplatelet treatment and CTR populations.

An important addition of our study is that we included HV and CTR, consisting in patients who were not undergoing antiplatelet treatment but had at least one cardiovascular risk factor. We found that inter-individual variability in platelet function is present in these patients, suggesting that a part of them present a hypo-reactive platelet phenotype, that could be potentially associated with consequent increased hemorrhagic risk and increased susceptibility to antiplatelet agents, while some others show a hyper-reactive platelet phenotype, that can be associated with increased thrombotic risk and reduced susceptibility to antiplatelet agents. This finding is of great interest, supporting the potential impact of implementing platelet reactivity and other markers of hypercoagulability in the stratification of individual patient’s risk of thrombotic events [38]. In addition, a recent genetic analysis identified 6 genes involved in platelet reactivity, with 4 of them involved in ADP-mediated platelet reactivity, that could allow to identify patients with hyper-reactive platelet phenotype at increased risk of thrombotic events that could benefit from antiplatelet treatment in primary prevention [39].

Finally, we found that CTRs had a more reactive platelet phenotype than HV, supporting the fact cardiovascular risk factors (i.e. diabetes mellitus, obesity, chronic kidney disease, advanced age) may significantly affect platelet reactivity and be at least in part responsible for the increased event rate observed in these patients [40, 41].

### Limitations

This study presents several limitations. First, it suffers from the limitations of observational studies and therefore its results should be considered as hypothesis generating. Second, we didn’t perform genetic testing for the cytochrome CYP2C19 which is responsible for the metabolism of clopidogrel. Therefore, our analysis could not have been stratified according to the individual ability to produce clopidogrel’s active metabolite. Third, because there is a substantial variability in the assessment of PA among different laboratories, data obtained in our laboratory could differ from that of other laboratories, preventing the direct comparison of our findings to that from other cohort of patients. However, the fact we used IQR to provide an objective estimation of OPR, HPR and LPR, and the inclusion of CTR and HV populations support the reliability of our findings.

Another limitation of the study is that platelet reactivity was not evaluated both before starting treatment and periodically throughout the course of treatment. Therefore, it remains unclear whether the high responsiveness of a particular patient is related to high responsiveness to the stimulant or to resistance to the drug.

## Conclusion

Our large database using a standardized approach based on the gold standard LTA suggests that there is a wide inter-individual variability in platelet function both in response to antiplatelet therapy, especially when single antiplatelet therapy is used, and in individuals with or without cardiovascular risk factors without antiplatelet therapy. The association between safety and efficacy outcomes with different degrees of platelet reactivity highlights the potential utility of using a strategy of guided escalation or de-escalation of antiplatelet therapy to optimize clinical outcomes.

## Electronic supplementary material

Below is the link to the electronic supplementary material.

Supplementary Material 1
