# Supplementary material for: Interindividual variability in platelet reactivity among individuals with or without antiplatelet therapy: results from a large tertiary care hospital
Source: J Thromb Thrombolysis. 2024 Sep 6;58(1):71–83. doi: 10.1007/s11239-024-03022-w (PMC11762593; doi:10.1007/s11239-024-03022-w)
Supplement: Supplementary file 1 — Supplementary Material 1 [file 11239_2024_3022_MOESM1_ESM.docx]

**SUPPLEMENTAL METHODS:**

**Patient information before test execution**

This is a retrospective study that examines a population that came to our attention for platelet aggregation tests, including 11913 patients from January 2004 to December 2022.

Population group allocation:

1. Healthy volunteers (HV) with no cardiovascular risk factors and any other diseases that haven’t received any kind of drugs in the 15 days before performing the analysis.
2. Control patients (CTR), patients with risk factors and taking medications to reduce the risk factors but who did not received any antiplatelet treatment.
3. Patients that were under acetylsalicylic acid treatment alone (ASA group), at low dosage (75-150 mg/die).
4. Patients under clopidogrel treatment alone (CLOP), at the dosage of 75mg/die.
5. Patients treated with double anti-aggregation therapy aspirin (75-150/die) plus clopidogrel (75 mg/die) (DAPT group).

Exclusion criteria are as follows:

a) Patients affected by platelet disorders, thrombocytosis, thrombocytopenia, platelet disease or

abnormal bleeding or diseases influencing platelet functioning (including hematological tumors,

HBV, HCV, HIV).

b) Patients who were under treatment with drugs acting on platelets and coagulation: Duoplavin,

Ibuprofen, Ibuprofen+ASA, Dipiridamole, Dipiridamole+ASA, Ibuprofen+Ticlopidine,

Ibuprofen+Dipiridamole, Picotamide, Ticlopidin, Ticlopidin+ASA, DOAC, VitK Inhibitor,

Heparin, Tranex, other antiplatelet (Prasugrel, Cilostazol, Ticagrelor), Non-Steroidal Anti-

Inflammatory Drugs (NSAIDs), Cortisone.

c) Patients that did not declared the correct therapy or suspended an antiplatelet drug for less than 7 days before the test.

Among the patients under antiplatelet treatment, we excluded those taking higher or lower antiplatelet dosage or reported whose administration was at alternate days.

The patients should be overnight fasting and does not take any occasional drugs during the week preceding the test. The biggest risk of a reduction in platelet response is represented by the action of drugs taken occasionally(1). The drugs mostly affecting platelet functionality are the anti-inflammatories, both steroidal and non-steroidal, whose wide occasional use is well known, for this reason a particular attention was given during the interview to drugs with antiplatelet action.

**Collection**

We carried out blood withdrawal avoiding any kind of stasis or turbulence.

For blood sampling we used butterfly needles 21G and for sample collection we used vacuette vacutainer 10 ml anticoagulated with 3.8% sodium citrate (Greiner Bio-One), with a 1:10 ratio (V:V).

**Transport**

Blood vials was maintained and carried at room temperature (RT), and in less than one hour platelet rich plasma (PRP) was prepared.

**Sample preparation**

Blood samples in sodium citrate, in 10 ml vials, were centrifuged at 200xg for 15 minutes at RT swing rotors to prepare PRP. The tube containing red blood cells and the leukocytes ring has been subjected to a further centrifugation for 10 minutes at 2000xg at RT, as to get a Platelet Poor Plasma (PPP).

Platelet aggregation has been evaluated in PRP in a four channel aggregometer (AggRam, Helena Laboratories, Beaumont, Texas, United States) according to the Born’s method(2).

If the instrument highlighted a high number of platelets we carried out a count of platelets in PRP by a red blood cell count and, where necessary (if higher than 500.000/mm^3^), PRP was diluted through a buffer solution at physiological pH(3).

We carried out the calibration of the instrument according to the indications reported in the user manual.

**Platelet agonists:**

For aggregometric studies we used ADP, epinephrine, collagen and arachidonic acid as agonists.

Herein are reported the agonists:

**ADP**

We used ADP at concentration 0.8 μM and 2 μM, if a secondary curves at 0.8 μM was obtained we used 0.4 μM and if there was not a secondary curve or platelets were not responsive at 2 μM we used the concentration 4 μM.

**COLLAGEN**

We used this agonist at a starting concentration of 2 μg/ml and if the platelet aggregation response was normal, 1 μg/ml concentration was tested. If PA at 2 μg/ml was low we tested collagen 4 μg/ml.

**EPINEPHRINE**

We used it at concentration 0.5 and 10 μM.

**ARACHIDONIC ACID (AA)**

Was used at concentration 0.5 mM in HV, CTR and CLOP patients, while it was tested AA 0.75 mM in ASA and DAPT patients.

**Agonist Preparation**

Platelet agonists were prepared according to the indications reported by the manufacturer.

**Platelet aggregation response: parameters**

The remarkable versatility of the test and its capability to highlight multiple phases of the platelet response, has brought to the existence of several variables that can be used to report the test results.

The PA parameter that we used is the platelet aggregation percentage (PA%) at 4 minutes. This parameter has the advantage to avoid errors due to maximum primary aggregations where it is not particularly clear whether the response is primary or secondary. Our database includes comprehensive records of both primary and secondary maximum aggregations obtained within 5 minutes of aggregation. To ensure that PA at 4 min parameter accurately reflects the platelet response to the agonist, we assessed the difference in aggregation at 4 minutes compared to the maximum aggregation (studied for 5 minutes), revealing that only 2% of the entire population shows a difference in aggregation percentage exceeding 10% for both the HV and CTR populations. These differences confirm the choice of 4-minute platelet aggregation as the great parameter for test evaluation.

**Supplement results**

The purpose of antiplatelet treatment is to reduce platelet functionality. To efficiently evaluate the pharmacological treatment, it is important to identify agonists concentration that can induce a high platelet response in most CTRs, at least a 75% PA. For this reason, among the concentrations used for all agonists, we presented only the results for PA% obtained in response to ADP 2 μM (70% CTR showed PA > 75%), collagen 2 μg/ml (97% CTR showed PA > 75%), adrenaline 10 μM (84% CTR showed PA > 75%), and arachidonic acid 0.5 (88% CTR showed PA > 75%) and for ASA and DAPT higher AA concentration was reported (0.75 mM), therefore the HPR can be defined at these values. Pharmacological treatment induces a PA reduction of less than 25% in a total of 13% in response to ADP, less than 1% for collagen, 5% for adrenaline, and 1% for AA, indicating that the treatment is very effective and therefore the LPR can be defined at these values.

In Table S1, we report the means±SD, medians and IQR of all the studied patients in response to various agonists. Furthermore, we report the outliers calculated according to the formulas respectively, for lower outlier = Q1 – (1.5 * IQR), and for higher outlier = Q3 + (1.5 * IQR), and the statistically significant differences between the CTR and individual populations, calculated using the Wilcoxon test.

**Table S1**

ADP 2 µM

| Populations (n) | Mean±SD | Median (IQR) | Outliers | Comparison  Vs. CTR |
| --- | --- | --- | --- | --- |
| HV (534) | 62.7±37.1 | 80 (35-93) | / | p< 0.0001 |
| CTR (1073) | 72.4±33.3 | 90 (62-94) | < 15% | / |
| ASA (3280) | 40.6±29.9 | 45 (0-65) | / | p<0.0001 |
| CLOP (495) | 25.1±35.1 | 0 (0-55) | / | p<0.0001 |
| DAPT (519) | 10.2±18.5 | 0 (0-13) | >31% | p<0.0001 |

Collagen 2 μg/ml

| Populations (n) | Mean±SD | Median (IQR) | Outliers | Comparison  Vs. CTR |
| --- | --- | --- | --- | --- |
| HV (531) | 90.7±11.6 | 93 (89-96) | < 80% | p=0.4789 |
| CTR (1072) | 90.7±10.5 | 92 (90-95) | < 83% | / |
| ASA (3225) | 40.8±26.3 | 36 (20-60) | / | p<0.0001 |
| CLOP (480) | 79.4±21.8 | 87 (80-90) | < 65% | p<0.0001 |
| DAPT (451) | 17.9±19.9 | 15 (0-28) | > 70% | p<0.0001 |

Epinephrine 10 μM

| Populations (n) | Mean±SD | Median (IQR) | Outliers | Comparison  Vs. CTR |
| --- | --- | --- | --- | --- |
| HV (420) | 78.6±28.1 | 90 (80-95) | < 57% | p=0.0587 |
| CTR (1073) | 82.5±23.4 | 90 (84-95) | < 68% | / |
| ASA (3225) | 32.1±30.8 | 40 (0-55) | / | p<0.0001 |
| CLOP (481) | 74.8±31.1 | 88 (75-92) | < 50% | p<0.0001 |
| DAPT (489) | 21.3±27.9 | 0 (0-47) | / | p<0.0001 |

Arachidonic acid (mM)

| ­ | mM | Mean±SD | Median  (IQR) | Outliers | Comparison  Vs. CTR |
| --- | --- | --- | --- | --- | --- |
| HV (369) | 0.5 | 87.6±20.5 | 93 (80-95) | < 80% | p< 0.0001 |
| CTR (978) | 0.5 | 90.7±15.6 | 92 (89-95) | > 0% | / |
| ASA (3138) | 0.75 | 2.6±12.6 | 0 (0-16) | / | p<0.0001 |
| CLOP (315) | 0.5 | 86.5±16.7 | 90 (87-93) | / | p<0.0001 |
| DAPT (377) | 0.75 | 4.3±9.4 | 0 (0-5) | / | p<0.0001 |

**Table S1:** Summary of statistical parameters for each studied population in response to ADP (2 µM), collagen (2μg/ml), epinephrine (10µM) and arachidonic acid (0.5 and 0.75 mM).

Values are reported as platelet aggregation percentage at 4 min through mean±SD, median (25-75% interquartile ranges). P-values test for differences between CTR population and antiplatelet treated populations. ASA, aspirin; CLOP, clopidogrel; CTR, control group; DAPT, double antiplatelet treatment; HV, healthy volunteers; IQR, interquartile range.

**References**

1. Kottke-Marchant K, Corcoran G. The laboratory diagnosis of platelet disorders. Arch Pathol Lab Med. 2002;126(2):133-46.

2. Born GVR. The aggregation of blood platelets by adenosine diphosphate and its reversal. Nature. 1962;194:927-9.

3. Cattaneo M, Lecchi A, Zighetti ML, Lussana F. Platelet aggregation studies: autologous platelet-poor plasma inhibits platelet aggregation when added to platelet-rich plasma to normalize platelet count. Haematologica. 2007;92(5):694-7.
